# Visfatin is a multifaceted molecule that exerts regulation effects on inflammation and apoptosis in RAW264.7 cells and mice immune organs

**DOI:** 10.3389/fimmu.2022.1018973

**Published:** 2022-12-01

**Authors:** Zhewei Zhang, Ke Xiao, Sheng Wang, Abdur Rahman Ansari, Xiaoyu Niu, Wenjie Yang, Mengqi Lu, Zhi Yang, Zia ur Rehman, Weihua Zou, Weicheng Bei, Hui Song

**Affiliations:** ^1^College of Animal Science and Veterinary Medicine, Huazhong Agricultural University, Wuhan, China; ^2^The Brain Cognition and Brain Disease Institute of Shenzhen Institutes of Advanced Technology, Chinese Academy of Sciences, Shenzhen, China; ^3^Section of Anatomy and Histology, Department of Basic Sciences, College of Veterinary & Animal Sciences, Jhang University of Veterinary and Animal Sciences, Lahore, Pakistan; ^4^Animal Health Supervision Institute of Taihe County, Fuyang, China; ^5^College of Veterinary Sciences, Faculty of Animal Husbandry and Veterinary Sciences, University of Agriculture, Peshawar, Pakistan; ^6^Wuhan Keqian Biology Company Limited, Wuhan, China

**Keywords:** visfatin, inflammation, apoptosis, immune organs, multifaceted molecule, FK866, RAW264.7 cells

## Abstract

Visfatin, a multifunctional adipocytokine, is particularly important in the regulation of apoptosis and inflammation through an unidentified mechanism. Clarifying the control mechanisms of visfatin on inflammation and apoptosis in RAW264.7 cells and mice immunological organs was the goal of the current investigation. In order to create a pathophysiological model, the RAW264.7 cells were stimulated with 200 ng/mL visfatin and 20 μg/mL lipopolysaccharide (LPS), either separately or combined. The effects of exogenous visfatin on inflammation and apoptosis in RAW264.7 cells were investigated by flow cytometry assay, RNA-seq analysis and fluorescence quantitative PCR. According to the findings, exogenous visfatin exhibits dual effects on inflammation by modulating the expression of IL-1α, TNFRSF1B, and LIF as well as taking part in various signaling pathways, including the MAPK and Rap1 signaling pathways. By controlling the expression levels of Bcl2l1, Bcl2a1a, and Fas and primarily participating in the PI3K/AKT signaling pathway and Hippo signaling pathway, exogenous visfatin can inhibit apoptosis in RAW264.7 cells. The visfatin inhibitor FK866 was used to further confirm the effects of visfatin on inflammation and apoptosis in mice immune organs. Subsequently, mice spleen and thymus were collected. It is interesting to note that in LPS-treated mice, suppression of endogenous visfatin might worsen the immune system’s inflammatory response and even result in rapid mortality. Additionally, endogenous visfatin promotes the apoptosis in mice immune organs by regulating the expression levels of Bcl2l1, Fas, Caspase 3, Bcl2a1a, and Bax. Together, these results imply that visfatin is a multifaceted molecule that regulates inflammation and apoptosis in RAW264.7 cells and mice immunological organs by taking part in a variety of biological processes and regulating the amounts of associated cytokines expression. Our findings offer additional understandings of how visfatin affects apoptosis and inflammation.

## Introduction

As an extremely significant endocrine organ of the body, adipose tissue not only stores energy, but also releases various adipokines in the body, including certain biologically active hormones and cytokines, which contributes to maintain the body’s homeostasis (1–3). The visfatin secreted by adipose tissue is an immune regulating cytokine, that may intricate in the inflammatory processes and is positively regulated in response to microbial stimulation by T cells, B cells, macrophages, neutrophils, and monocytes (4, 5). Visfatin acts a key mediator between inflammation and innate immune response (6, 7) and regulates the synthesis of certain inflammatory cytokines (8), suggesting its key role in acquired as well as in the innate immune response. Visfatin is an essential factor associated with inflammation, immune stress, cell proliferation, apoptosis, and autophagy (9–12). Externally added visfatin has ability to activate various pathways such as NF-κB, phosphatidylinositol 3 kinase (13, 14), MAPK-related pathways (15–18).

Several reports showed anti-apoptotic effects of visfatin in different cell types, such as B cells, T cells, macrophages, neutrophils, and vascular SMCs. Moreover, both intracellular and extracellular visfatin were found to promote these effects (19–22). Furthermore, visfatin can prevent macrophages from programmed cell death and promote macrophage survival rate *via* a non-enzymatic IL-6/STAT3 signaling mechanism (23). A growing number of studies stated that visfatin plays a key role in sustaining the homeostasis of the body’s physiological state and inflammatory reaction by regulating immune response, inflammation, cell proliferation, autophagy, apoptosis, and necrosis (24–26). Moreover, visfatin treatment not only reduces inflammation, apoptosis and necrosis but also regulates LPS-induced apoptosis and inflammation by playing a major role in mitochondrial pathway-mediated apoptosis and inflammation (27, 28).

Earlier studies have presented that visfatin may exhibit a dual regulation effect on cell apoptosis in rat spleen, and synergistic regulation of visfatin on autophagy and apoptosis in mice inflammatory model (14, 29). However, the functional mechanism of visfatin on inflammation and apoptosis is not fully clarified yet. Our current study was performed to further explore the regulation effects of exogenous and endogenous visfatin on inflammation and apoptosis in RAW264.7 cells and mice immune organs. Furthermore, it is a novel study that created abundant gene expression data facilitating further comprehensive studies regarding the mechanisms of action of visfatin, thereby clarifying the biological processes and important signaling pathways involved in the regulation of inflammation and apoptosis by visfatin.

## Materials and methods

### Reagents

Lipopolysaccharide (LPS) *E.coli* O111:B4 was obtained from Sigma Aldrich (St. Louis, MO, USA). Recombinant mouse visfatin was obtained from Prospec (cyt-447, Prospec, Germany). FK866 was procured from Beyotime Institute of Biotechnology (Shanghai, China). The antibody of visfatin for immunohistochemistry (IHC) was obtained from ABClonal (Wuhan, China). Horseradish peroxidase (HRP) Conjugated Goat Anti-Mouse IgG was purchased from Yifeixue Bio Tech (Nanjing, China).

### Cell culture

Murine macrophage RAW264.7 cells (ATCC^®^ TIB-71™) was grown in 25 cm^2^ culture flasks (Corning, Sigma-Aldrich) having Dulbecco’s modified Eagle’s medium (DMEM) augmented with 10% fetal calf serum, L-glutamine (2 mM) and antibiotics (penicillin, 100 U/mL & streptomycin, 100 U/mL) in an incubator (5% CO_2_) at 37°C in humidified atmosphere. RAW264.7 cells were sub-cultured into new flasks containing fresh culture media thrice weekly.

RAW264.7 cells were allocated into 4 groups with 6 replications in each group: Control group, LPS group, Visfatin group and LPS+visfatin group. All the chemicals used in different treatments were pre-dissolved with DMEM. Complete medium was added in Control group, complete medium containing 200 ng/mL visfatin was incorporated in Visfatin group, complete medium having 20 μg/mL LPS was added in LPS group and medium containing 200 ng/mL visfatin and 20 μg/mL LPS was added in LPS+visfatin group. Sampling was performed at 6, 12, 18, 24 h from these treated cell culture.

### Cell viability assay

The 3-(4,5-dimethyl-2-thiazyl)-2,5-diphenyl-2H-tetrazolium-bromide (MTT) assay was used to determine the viability. Concisely, the RAW264.7 cells (1 × 10^6^ cells/mL) were seeded in 96-well microplates in 100 μL per well for 12 h. Subsequently, the cells were treated with numerous concentrations of visfatin and LPS for 24 h. The final volume was 200 μL. A control group and a medium alone group were also included. Then, the medium was removed while MTT solution (20 μL, 5 mg/mL; Sigma Aldrich, USA) was added for 4 h and the reaction was terminated by the addition of 100 μL Dimethyl sulfoxide (DMSO). The formazan crystals were dissolved in DMSO (100%). The absorbance values were taken at 570 nm using a microplate reader (Model 550, BioRad Lab, USA). The viability was measured using the formula: percent of viable cells = (absorbance of experimental wells/absorbance of control cells) × 100%.

### Flow cytometry

An annexin V-FITC/PI apoptosis detection kit was used to measure the phosphatidylserine exterior of the apoptotic cells. Briefly, RAW264.7 cells were treated with DMEM, LPS, visfatin and LPS+visfatin for 6, 12, 18, and 24 h, earlier they were collected and stained with Annexin V and PI by an annexin V-fluorescein isothiocyanate (FITC)/Propidium Iodide (PI) kit (KeyGEN, Nanjing, China) for cytometry analysis. All the experiments were accomplished in triplicates and the data demonstration at least three independent experiments.

### RNA isolation and qRT-PCR

Total RNA was extracted from RNAW264.7 cells with the TRIzol reagent (Invitrogen) as instructed by manufacturer. Using NanoDrop 2000 spectrophotometer (Thermo Scientific) RNA was quantified. RNA was transcribed to cDNA with the PrimeScript™ RT Reagent Kit (Takara, Clontech). Contaminating genomic DNA was degraded using gDNA Eraser (Takara, Clontech).

The primers were designed using Primer 3. The mice primer sequences for β-actin, Fas, LIF, IL-6, TNF-α, IL-1α, IL-1β, Caspase3, Bcl2l1, Bcl2a1a, Bax, and TNFRSF1B were listed in **Table 1**. qRT-PCR was performed on a CFX384 (BioRad, USA) with SYBR^®^ Premix Ex Taq^TM^ II (Takara, Clontech). PCR reactions were as follows: activation at one cycle of 50°C for 2 min, 95°C for 3 min, followed by 33 cycles of 95°C for 30 s and 58°C for 30 s. β-actin was used as a reference gene to calculate the relative abundance of the target genes and the qRT-PCR data were analyzed by a comparative threshold method.

**Table 1:** Mice Primer Sequences for qRT-PCR.

Gene	Forward primer	Reverse primer
β-actin	5’-CACTGCCGCATCCTCTTCCTCCC-3’	5’-CAATAGTGATGACCTGGCCGT-3’
Fas	5’-CAAGTGCAAGTGCAAACCAG-3’	5’-GGGTTCCATGTTCACACGA-3’
LIF	5’-GGGATTGTGCCCTTACTGCTG-3’	5’-AGAAGGCCTGGACCACCACACT-3’
IL-6	5’-ACTTCCATCCAGTTGCCTTC-3’	5’-ATTTCCACGATTTCCCAGAG-3’
TNF-α	5’-CCGACCTCATTTTCTTCTGG-3’	5’-CACTTGGTGGTTTGCTACGAC
IL-1α	5’-TCTTCTCATTCCTGCTTGTGG-3’	5’-CCGACCTCATTTTCTTCTGG-3’
IL-1β	5’-TGACGGACCCCAAAAGATGA-3’	5’-TCTCCACAGCCACAATGAGT-3’
Caspase3	5’-GAGCTTGGAACGGTACGCTA-3’	5’-GAGTCCACTGACTTGCTCCC-3’
Bcl2l1	5’-GAGAGGCAGGCGATGAGTTT-3’	5’-CCCCAGTTTACTCCATCCCG-3’
Bcl2a1a	5’-GAGTTGCTTTCTCCGTTCAG-3’	5’-GTCACAATCCTTCCCCAGTT-3’
Bax	5’-CGTGAGCGGCTGCTTGTCTG-3’	5’-ATGGTGAGCGAGGCGGTGAG-3’
TNFRSF1B	5’-CAAGGGTGGCATCTCTCTTC-3’	5’-GCATCTCTTTGTAGGCAGGA-3’

### RNA-seq library preparation and RNA-seq data analysis

RAW264.7 cells were treated with DMEM, LPS, visfatin and LPS+visfatin, then harvested at 12 h and resuspended in Trizol (Invitorgen). mRNA was extracted from total RNA using capture beads (Vazyme). For synthesis of double-stranded cDNA from the purified polyadenylated mRNA templates, the PrimeScript^TM^ Double Strand cDNA Synthesis Kit (Takara) was used. cDNA libraries for Illumina sequencing were prepared using the TruePrep DNA library Prep Kit V2 for Illumina (Takara).

Differentially expressed genes (DEGs) were evaluated by HTSeq software among the control, LPS, visfatin, and LPS+visfatin libraries after pre-processing. Further data mining was accomplished including: DEGs analysis by DESeq2 package with default parameters, gene ontology (GO) analysis by DAVID and cluster profiler package. Furthermore, analysis was conducted using GO plot with the results from DAVID, which all are available in R packages (30).

### Animal experiments and visfatin treatment

All mice (Male Kunming mice, 6 weeks old, 20 ± 2 g) were purchased from the Hubei Province Disease Control Center (China). The Scientific Ethic Committee of Huazhong Agricultural University approved animal experiments (HZAUMO-2019-025). Forty animals were divided into four groups with 10 mice in each group. The Control group and the FK866 group received single-dose injections of isotonic saline (0.2 ml, i.v.) and FK866 (10 mg/kg bw, i.v.) for 7 consecutive days. The LPS group received a single-dose injection of LPS (8 mg/kg bw, i.v.) all at once for the establishment of a pathological model in mice, and the LPS+FK866 group received single doses of FK866 (10 mg/kg bw, i.v.) for 7 consecutive days following a single-dose injection of LPS (8 mg/kg bw, i.v.). Mice were housed in a temperature-controlled and light-controlled room (12 h light/12 h dark; 23-25°C) and were allowed ad libitum access to tap water and standard commercial mouse food. All mice were anesthetized with 1% pentobarbital (25 mg/kg bw, i.v.) in tail veins. The animals were euthanized at the end of the experiments or when moribund during the experiment. Subsequently they were sacrificed 12 h after the last treatment and the spleen and thymus were removed from each mouse. Part of the tissues were frozen in liquid nitrogen and another part was fixed in 4% buffered paraformaldehyde solution. In another test, 20 animals were divided into two groups with 10 mice in each group. The FK866 group received single-dose injections of FK866 (10 mg/kg bw, i.v.) for 7 consecutive days, while the LPS+FK866 group received single doses of FK866 (10 mg/kg bw, i.v.) for 7 consecutive days following a single lethal dose of LPS (20 mg/kg bw, i.v.). After 5 days of continuous observation, the survival rate of mice in each group was counted, and the animals were euthanized at the end of the experiments or when moribund during the experiment.

### Histologic analysis of mice spleen and thymus

The spleen and thymus samples of each mouse were fixed with 4% paraformaldehyde, embedded in paraffin, and then cut into 3-5 μm thick sections. Hematoxylin-eosin (HE) staining was done using routinely used protocol. Briefly, the spleen and thymus tissue sections were stained with hematoxylin for 8 min, treated with l% hydrochloric acid (HCl) for 6-8 s and finally stained with eosin for 1 min. Moreover, the expressions of visfatin were detected by IHC according to the manufacturer’s instructions. IHC staining was evaluated by high-power light microscopic examination (BX51; Olympus, Tokyo, Japan), and the integrated option density (IOD) was statistically analyzed by Image Pro Plus 6.0 (Media Cytology, Inc).

### Statistical analysis

All data were presented as the mean ± SD (Standard Deviation) of independent measurements. One-way analysis of variance (ANOVA, LSD test) was used to evaluate the significance of differences in different groups. Statistical analysis was performed with GraphPad Prism 5.0 (GraphPad Software, San Diego, CA). Significance levels were set at *P*<0.05 or *P*<0.01.

### Data availability

The raw sequencing data from this study are available from the NCBI Short Read Archive (SRA) with the following accession numbers (PRJNA293603). https://www.ncbi.nlm.nih.gov/bioproject/293603

## Results

### Screening of the optimal dose of visfatin and LPS in RAW264.7 cells

RAW264.7 cells were first exposed to different concentrations of visfatin and LPS for 24 h, and then the optimal dose of each substance was determined by evaluating cell viability and morphology. The results indicated that cells of untreated Control group were mostly smooth, without pseudopodia, the cell morphology did not change significantly after treatment with different concentrations of visfatin, whereas those stimulated with different concentrations of LPS showed characteristics of activation of macrophages, such as rounding of cells, shrinkage, and increased pseudopodia.

**Figure 1:**
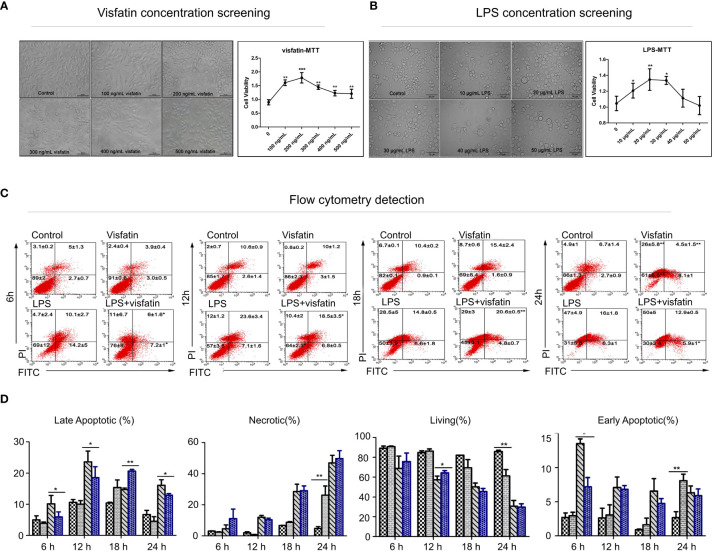
Effects of visfatin on cell proliferation and apoptosis in RAW264.7 cells. **(A)** Screening of the optimal dose of visfatin acting on RAW264.7 cells (**P<0.01). RAW264.7 cells were treated with numerous concentrations of visfatin for 24 h, and the viability was assessed by MTT assay. **(B)** Screening of the optimal dose of LPS stimulated RAW264.7 cells (*P<0.05, and **P<0.01). RAW264.7 cells were treated with numerous concentrations of LPS for 24 h, and the viability was assessed by MTT assay. **(C)** Flow cytometry to detect the role of visfatin in different stages of cell apoptosis (*P<0.05, and **P<0.01). RAW264.7 cells were treated with visfatin, LPS, and LPS+visfatin for different time intervals from 6 h to 24 h. **(D)** The ratios of live, early apoptotic, late apoptotic, and necrotic cells after exposure to visfatin, LPS, and LPS+visfatin for various time intervals.

Meanwhile, the changes in cell viability after different treatments were identified by MTT, and the outcomes showed that, following treatment with various concentrations of visfatin, cell viability dramatically increased, reaching its greatest level at a visfatin concentration of 200 ng/mL (*P*<0.01; **Figure 1A**). Similarly, the cell viability was highest when cells were stimulated by 20 μg/mL LPS, while an excessive amount of LPS dramatically reduced cell viability (*P*<0.05, *P*<0.01; **Figure 1B**). Therefore, cells in Visfatin group were treated with 200 ng/mL visfatin, cells in LPS group were treated with 20 μg/mL LPS, while cells in LPS+visfatin group co stimulated with LPS and visfatin at the above dose for the subsequent experiments.

### Effects of exogenous visfatin on apoptosis in RAW264.7 cells

The Control group, Visfatin group, LPS group and LPS+visfatin group were established, and by using flow cytometry, the apoptosis was found in each group at various times. The values from the flow cytometry assay were listed in **Supplementary Table 1**. At 6 h, LPS stimulation dramatically accelerated both the early and late stages of apoptosis. As opposed to the LPS group, apoptosis was markedly reduced in the LPS+visfatin group.

When both early and late apoptosis in RAW264.7 cells were significantly increased, while the late apoptotic stage in cells co-treated with LPS+visfatin was markedly downregulated compared to those in the LPS group and the early apoptotic stage in LPS+visfatin group was slightly lower than that of LPS group, meanwhile the cell survival rate was considerable increased at 12 h. The survival rate of the cells in each group declined steadily as treatment duration increased. Visfatin significantly boosted early apoptosis and necrosis in cells compared to the Control group at 24 h, and LPS+visfatin considerably reduced late apoptosis compared to the LPS group (*P*<0.05, *P*<0.01; **Figures 1C, D**). In order to conduct further research, visfatin and LPS therapy for 12 h was chosen. Consequently, the aforementioned findings first supported exogenous visfatin’s ability to prevent LPS-induced apoptosis in RAW264.7 cells.

### Effects of visfatin on inflammation and apoptosis in RAW264.7 cells detected by RNA-seq analysis

**Figure 2:**
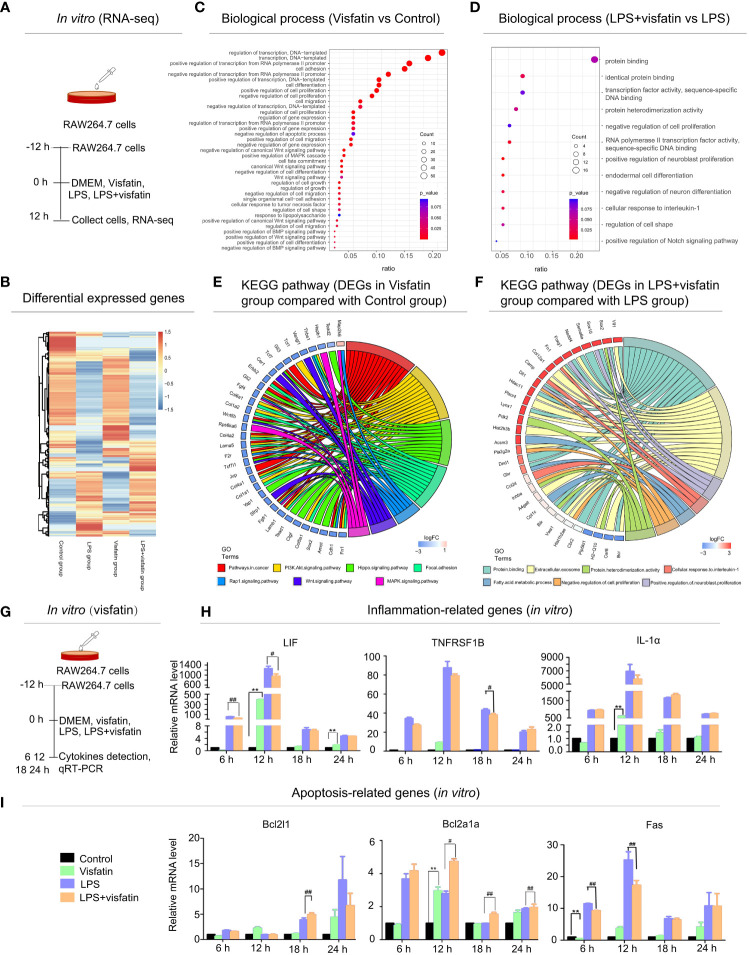
Effects of exogenous visfatin on inflammation and apoptosis in RAW264.7 cells detected by RNA-seq analysis. **(A)** Treatment of RAW264.7 cells in different ways for RNA-seq analysis after 12 h (*in vitro*). **(B)** Heatmap of regulated DEGs between Control, Visfatin, LPS and LPS+visfatin in four groups illustrating the gene expression patterns. **(C, D)** Gene ontology analysis of regulated DEGs were displayed for Visfatin vs Control and LPS+visfatin vs LPS groups, enriched terms were ranked by gene ratio and -log10 (*P* value). **(E, F)** GO plots were used for seven most significant pathways and biological processes for Visfatin vs Control, LPS+visfatin vs LPS group. Associated DEGs were also checked to reveal the reaction after treatment with LPS. **(G)** *In vitro* verification of RNA-seq analysis results. **(H, I)** Detection of inflammatory factors and apoptosis-related cytokines in RAW264.7 cells of different treatment groups (compared with the control group: **P<0.01; compared with the LPS group: ^#^*P*<0.05, ^##^*P*<0.01).

For further confirmation of the role of exogenous visfatin on inflammation and apoptosis in RAW264.7 cells. RAW264.7 cells were divided into 4 groups by different treatments and harvested at 12 h after treatment, then cellular RNA was extracted and a cDNA library was constructed for RNA-seq analysis (**Figure 2A**). A total of 1225 DEGs were found, of which 692 were up-regulated and 527 were down-regulated, according to the heatmap diagram (**Figure 2B**). Deeper functional annotation of genes derived from different groups were further performed by analyzing the biological processes and pathways involved in the identified DEGs.

According to gene ontology (GO) data, the up-regulated DEGs in the Visfatin group were significantly enriched in biological processes such as transcription regulation, cell proliferation, differentiation, and apoptosis, cell adhesion, migration and inflammation when compared to the Control group (**Figure 2C**). Additionally, in comparison to the LPS group, the LPS+visfatin group’s up-regulated DEGs were primarily linked to biological processes such as protein binding, cell shape, differentiation, proliferation, and cellular response to interleukin-1, as well as the Notch signaling pathway (**Figure 2D**).

In order to elucidate the main signaling pathways and key genes involved in the regulation of visfatin in cell pathophysiological conditions, apoptosis and inflammation were screened out and analyzed in combination with their biological processes and related DEGs. Circos graph result indicated that a total of 7 signaling pathways were screened between the Visfatin and the Control groups. Combined with the analysis of these seven important biological processes and their related DEGs, the findings revealed that 34 genes with variable expression were linked to changes in the related biological pathways and activities (**Figure 2E**). In addition, a total of 7 signaling pathways were screened in LPS+visfatin and LPS groups. These biological processes contain 31 DEGs (**Figure 2F**).

*In vitro* testing confirmed the existence of the genes involved in inflammation and apoptosis. RAW264.7 cells were separated into four treatment groups, and samples were taken at intervals of 6 h, 12 h, 18 h and 24 h for the purpose of cytokine detection (**Figure 2G**). LIF, TNFRSF1B, and IL-1α mRNA expression levels were elevated in the Visfatin group at various time points, while LIF and IL-1α expressions were substantially higher in the Visfatin group than the Control group at 12 h. When compared to the LPS group, the LPS+visfatin group’s LIF decreased at various time points, with the most significant decreases occurring at 6 h and 12 h. Meanwhile, the TNFRSF1B reduced at both 6 h and 12 h, with the most significant decrease occurring at 18 h. Additionally, at 6 h and 12 h, the mRNA expression levels of IL-1 dramatically reduced, whereas at 18 h and 24 h, they slightly increased (*P*<0.01; **Figure 2H**).

Additionally, the pro-apoptotic gene Fas was noticeable decreased by about 25% and 36% in the LPS+visfatin group as compared to the LPS group at 6 and 12 h, whereas a significant increase of mRNA expression levels by about 31% for Bcl2l1 at 18 h and approximately 53%, 60% and 9% for Bcl2a1a at 12, 18, 24 h, respectively (*P*<0.01; **Figure 2I**), which was demonstrated that visfatin may exhibit dual regulation effects on apoptosis and inflammation in healthy and diseased situations.

### Effects of endogenous visfatin on the pathological morphology of immune organs and survival rate in LPS-treated mice

**Figure 3:**
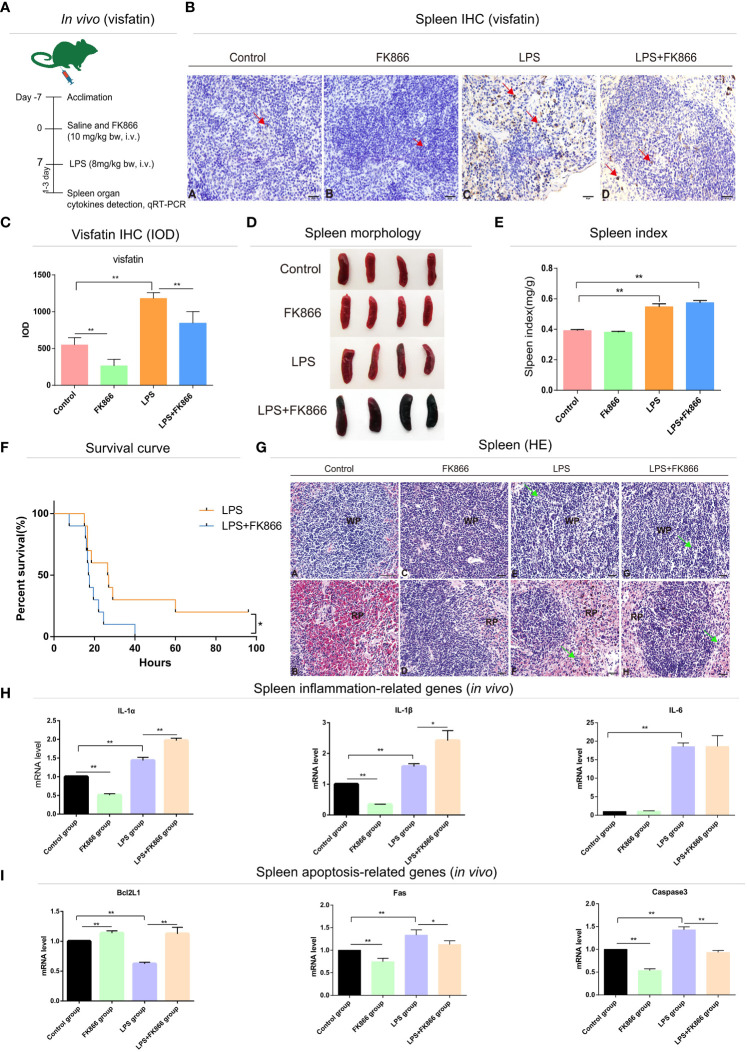
Effects of endogenous visfatin on inflammation and apoptosis in mice spleen. **(A)** Experimental timeline of the exogenous FK866 (10 mg/kg bw, i.v.) and LPS (8 mg/kg bw, i.v.) treatment in mice (*n*=10 per group, *in vivo*). **(B)** IHC detection of visfatin expression in mice spleen from different treatment groups, Yellowish brown reaction product refers to visfatin-positive signal distribution, marked by red arrow. **(C)** Detection of the IOD value and counting of the expression of visfatin in mice spleen from different treatment groups (**P<0.01). **(D)** Gross lesions of the mice spleen in different treatment groups. **(E)** Spleen index of different treatment groups (**P<0.01). **(F)** Survival rates of different treatment groups (*P<0.05). The test was divided into two groups, the FK866 group received single-dose injections of FK866 (10 mg/kg bw, i.v.) for 7 consecutive days, while the LPS+FK866 group received single doses of FK866 (10 mg/kg bw, i.v.) for 7 consecutive days following a single lethal dose of LPS (20 mg/kg bw, i.v.). Statistics of survival of mice in each group (*n*=10 per group). **(G)** Histological analysis mice spleen in different treatment groups. WP, white pulp; RP, red pulp; Green arrows point to apoptotic bodies. Spleen WP (A) and RP (B) in control group; spleen WP (C) and RP (D) in FK866 group; spleen WP (E) and RP (F) in LPS group; spleen WP (G) and RP (H) in LPS+FK866 group. **(H)** qRT-PCR detection of the mRNA expression levels in inflammation-related cytokines of the mice spleen in different treatment groups (*P<0.05, and **P<0.01). **(I)** qRT-PCR detection of the mRNA expression levels in apoptosis-related cytokines of the mice spleen in different treatment groups (*P<0.05, and **P<0.01).

**Figure 4:**
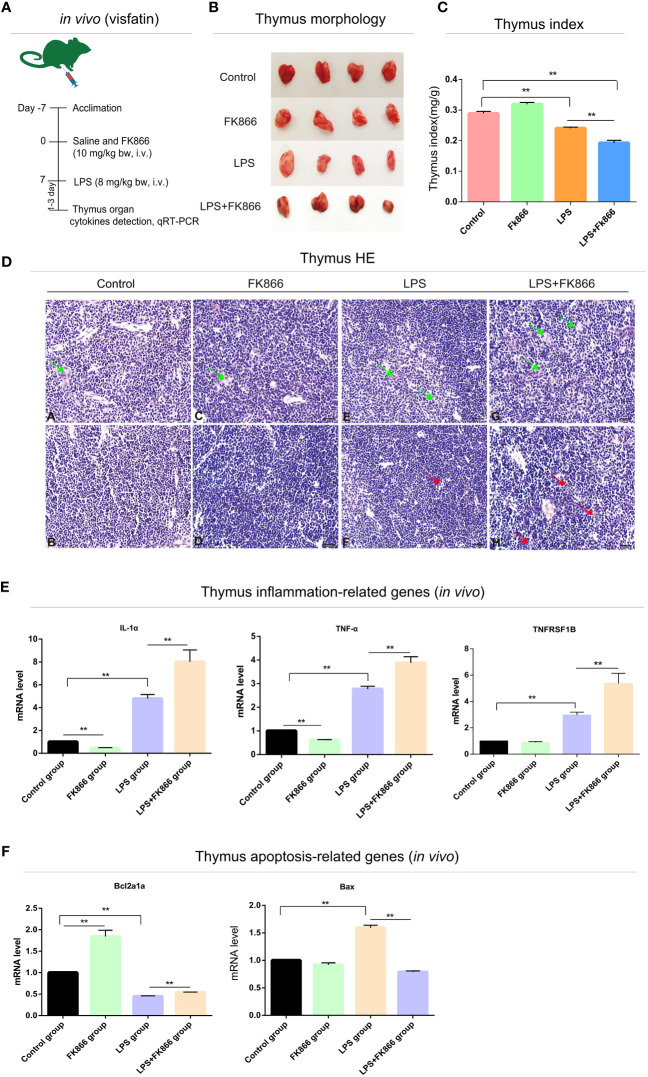
Effects of endogenous visfatin on inflammation and apoptosis in mice thymus. **(A)** Experimental timeline of the exogenous FK866 (10 mg/kg bw, i.v.) and LPS (8 mg/kg bw, i.v.) treatment in mice (*n*=10 per group, *in vivo*). **(B)** Gross lesions of the mice thymus in different treatment groups. **(C)** Thymus index of different treatment groups (**P<0.01). **(D)** Histological analysis mice thymus in different treatment groups. Green arrows point to thymic bodies, red arrows point to apoptotic bodies. Thymus medulla (A) and cortex (B) in control group; Thymus medulla (C) and cortex (D) in FK866 group; Thymus medulla (E) and cortex (F) in LPS group; Thymus medulla (G) and cortex (H) in LPS+FK866 group. **(E)** qRT-PCR detection of the mRNA expression levels in inflammation-related cytokines of the mice thymus in different treatment groups (**P<0.01). **(F)** qRT-PCR detection of the mRNA expression levels in apoptosis-related cytokines of the mice thymus in different treatment groups (**P<0.01).

To explore the effects of endogenous visfatin on the structure of immune organs in LPS-treated mice. The visfatin inhibitor FK866 was pre-injected into mice to inhibit the expression of endogenous visfatin in mice (31) (**Figures 3A**, **4A**). Visfatin expression levels were measured by IHC in mice spleen. The results showed that visfatin-positive cells were significantly less in FK866 group as compared to the Control group, but significantly more in the LPS and LPS+FK866 groups, while exceedingly decreased in LPS+FK866 group compared to the LPS group (*P*<0.01; **Figures 3B, C**). This research showed that FK866 injection decreased the endogenous visfatin expression in mice. Furthermore, we discovered no abnormalities in the Control and FK866 groups. The spleen samples were slightly enlarged, blackened, and hemorrhagic in the LPS group, while this condition was worse in the LPS+FK866 group (**Figure 3D**). In both the LPS and LPS+FK866 groups, hemolyzed erythrocytes were found in the red pulp along with a dramatic drop in lymphocytes. Additionally, many lymphocytes were observed with pyknotic nuclei and death of lymphocytes to form apoptotic bodies, which were more apparent in LPS+FK866 group (**Figure 3G**). Moreover, the thymus in the LPS+FK866 group was highly atrophied and necrotic in comparison to the LPS group (**Figure 4B**), and numerous thymic bodies and apoptotic bodies could be seen in LPS+FK866 group (**Figure 4D**).

As shown in **Figure 3E**, compared to the Control group, the spleen index of the mice in LPS and LPS+FK866 groups increased with high significance, while slightly higher in LPS+FK866 group than that in LPS group. However, the thymus index was significantly reduced in LPS+FK866 group as compared to the LPS group (**Figure 4C**). Additionally, our data demonstrated that the percentage of mice in the LPS+FK866 group that survived was significantly lower than that of the mice in the LPS group (*P*<0.05; **Figure 3F**).

### Effects of endogenous visfatin on inflammation and apoptosis in mice immune organs

Effects of endogenous visfatin on inflammation and apoptosis of immune organs were further confirmed by qRT-PCR. The mRNA expression levels of the inflammation-related genes IL-1α and IL-1β of mice spleen in the FK866 group were significantly lower than those in the Control group, but they were significantly higher in the LPS+FK866 group when compared to the LPS group. Additionally, compared with the Control group, the mRNA expression level of IL-6 increased significantly in LPS group, which was equivalent to that in LPS+FK866 group (*P*<0.01; **Figure 3H**). Furthermore, similar results were also found in the thymus of mice in each group. Compared with Control group, the mRNA expression levels of IL-1α and TNF-α were significant decreased in FK866 group, however the expression level of TNFRSF1B was marginally lower. Following LPS stimulation, IL-1α, TNF-α and TNFRSF1B mRNA expression levels were significantly higher than those in Control group, while IL-1α, TNF-α and TNFRSF1B mRNA expression levels in LPS+FK866 group increased significantly when compared to the LPS group (*P*<0.01; **Figure 4E**).

We evaluated the levels of expression of cytokines associated with apoptosis in mice immune organs in addition to the inflammatory cytokines mentioned above. The findings showed that the mRNA level of anti-apoptotic gene Bcl2l1 was significantly increased about 13% in the FK866 group when compared to the Control group, but remarkable decreased nearly 37% in the LPS group. However, the mRNA level of Bcl2l1 in the LPS+FK866 group was significantly up-regulated at approximately 78% than that in the LPS group. In contrast, the mRNA levels of the pro-apoptotic genes Fas and Caspase3 dramatically increased by nearly 32% and 40% in the LPS group while significantly decreasing by about 25% and 47% in the FK866 group. Additionally, a considerably lessened by approximately 11% for Fas and by nearly 36% for Caspase3 in LPS+FK866 group as compared to LPS group (*P*<0.01; **Figure 3I**). For the thymus samples, the mRNA expression level of anti-apoptotic gene Bcl2a1a in FK866 group was also significant increased by nearly 84% compared to the Control group, but noticeable decreased by about 55% in the LPS group. In contrast, the mRNA expression level of Bcl2a1a in the LPS+FK866 group was significant increased by about 20% than that in the LPS group. However, the mRNA level of pro-apoptotic gene Bax was considerably increased by nearly 59% in the LPS group while it decreased in the FK866 group. Additionally, the LPS+FK866 group’s Bax mRNA level was remarkably reduced by approximately 53% than that of the LPS group (*P*<0.01; **Figure 4F**).

## Discussion

### Effects of exogenous visfatin on cell proliferation in RAW264.7 cells

The current investigation determined how exogenous visfatin affects cell proliferation utilizing an *in vitro* model using LPS-elicited RAW264.7 cells. According to the findings, visfatin treatments at various concentrations might greatly increase cell survival without changing shape. Cell viability peaked at a visfatin concentration of 200 ng/mL and an LPS concentration of 20 μg/mL. Cells treated with LPS displayed macrophage activation characteristics, and excessive amounts of LPS gradually decreased cell viability. Numerous investigations have shown that visfatin is directly related to cell proliferation, supporting our findings. Recombinant visfatin boosted cell proliferation and DNA synthesis rate in human breast cancer cells when it was administered exogenously (MCF-7) (32). Moreover, visfatin might play a vital role in hepatocellular carcinoma cells (HCC) proliferation and be related with the progression of this malignancy (33). In addition, the proliferation of cardiac fibroblasts cells (CFs) was significantly promoted by visfatin in a dose dependent manner (34).

### Visfatin regulates inflammation and apoptosis by primarily participating in MAPK, Rap1, PI3K/AKT, and Hippo signaling pathways

Through RNA-seq research, we discovered in the current study that visfatin primarily maintains physiological processes such differentiation, cell proliferation, adhesion, and migration in healthy cell states. Visfatin is also found to be involved in the maintenance of cell inflammation, apoptosis, and the response to LPS stimulation. Additionally, it was discovered that visfatin is engaged in both physiological and inflammatory states of the body by screening out many signaling pathways.

Previous studies established that visfatin influences a variety of physiological processes, including differentiation and cell proliferation (35–37), hence supporting our current findings. Visfatin also plays a significant role in the development of tumors by taking part in the control of many signaling and cancer-related pathways. Many studies demonstrated the role of visfatin in metastasis and angiogenesis of breast cancer (32). Another study indicated that visfatin promoted the proliferation of HCC cells and might be linked with the progression of this malignancy. Moreover, branched chain amino acid (BCAA) might inhibit obesity-related liver carcinogenesis by reducing stimulatory effect of visfatin (38).

The MAPK signaling pathway is believed to regulate various essential physiological and pathological effects such as differentiation, cell growth, stress, and inflammatory responses (39). MAPK-p38 not only regulates the inflammatory responses (40), but also facilitates the phagocytic activity in LPS-stimulated macrophages (41). Additionally, the PI3K/AKT intracellular signaling pathway is associated with proliferation, cellular quiescence, cancer, and longevity. Moreover, PI3K lead to phosphorylation and further activation of Akt, which localizes to the cellular membrane (42, 43). PI3K/AKT signaling pathway activation during lung injury can both slow down the rate of apoptosis in alveolar type II cells and block the development of the Bax protein, which is generally implicated in lung protection (44). Similarly, Hippo signaling pathway was thought to have this effect as well (45). Sufficient evidence suggested that Rap1 signaling pathway is closely related to inflammatory reaction (46, 47).

### The regulation effects of exogenous visfatin on inflammation in RAW264.7 cells

An array of defensive measures taken by the host against external and internal dangers is known as inflammation (48). The inflammation is strongly regulated process and macrophages are involved in its commencement, continuation and resolution that synthesize many biologically mediators for both harmful and useful effects during inflammatory process (49, 50). In this study, 20 μg/mL LPS was used to stimulate RAW264.7 cells to activate macrophages. Meanwhile, the LPS+visfatin co-treatment group was set up to investigate the effect of exogenous visfatin on inflammation in RAW264.7 cells. Our findings showed that visfatin treatment of cells enhanced the levels of mRNA expression of inflammatory factors such as LIF, TNFRSF1B and IL-1α, indicating that visfatin has a pro-inflammatory function in cell normal state. However, visfatin could inhibit the mRNA expression levels of above cytokines when the cells were stimulated by LPS. An association between visfatin and inflammatory cytokines, such as TNF-α, IL-6, IL-8, IL-1β and IL-1α has been described in human fetal membranes and peripheral blood cells (51–53).

### The regulation effects of endogenous visfatin on inflammation in mice immune organs

We further verified the effect of endogenous visfatin on inflammation in mice immune organs. Mice were treated with FK866, a specific non-competitive NAMPT inhibitor, exerts significant inhibition of visfatin expression (54, 55). In this study, the expression level of visfatin in mice was significantly reduced after FK866 treatment. After LPS stimulation, a severe inflammatory response broke out in the mice. Histological staining results showed that LPS treatment caused different degrees of histological damage to the spleen and thymus, and the pathological damage was more obvious in the LPS+FK866 group. The LPS+FK866 group’s spleen organ index was marginally higher than that of the LPS group, while the thymus organ index was considerably lower than that of the LPS group. These findings show that the spleen, an adaptive immune organ, will enlarge and necrotize when there is significant inflammation, whereas the thymus, an innate immune organ, will acutely atrophy and necrotize when there is excessive inflammation. (56, 57).

Further examining the inflammatory factor expression levels in mice, we discovered that the expression of visfatin was inhibited and the expression level of inflammatory factors was reduced in FK866 group, indicating that visfatin might contribute to inflammation in the body’s normal state. The earlier observations exhibited that visfatin encourages the generation of circulating IL-1β, IL-6, TNF-α, IL-10 and IL-1α, thus these observations are consistent with the outcome of present study (6, 58). By stimulating the p38-MAPK signaling pathway, visfatin can boost the production of TNF-α, IL-6, IL-8, and MMP-9 in monocytes (59). Additionally, the results of the present investigation showed that a decrease in visfatin expression worsens the inflammatory response and is accompanied by an increase in the expression of a number of inflammatory cytokines such as IL-1α, IL-1β, TNF-α, and TNFRSF1B. This supports our theories and earlier studies that indicated visfatin might raise levels of both pro-inflammatory and anti-inflammatory cytokines including IL-6, TNF-α, IL-1β, IL-10 and IL-4 in rat spleen while inhibiting IL-6, IL-1β, IL-10 and IL-4 expression when LPS was present (14).

Notably, we found that the mice inhibited the expression of endogenous visfatin and all the mice died very quickly when stimulated by lethal dose of LPS. Lots of research showed that excessive inflammation might result in mortality by damaging tissues and organs in severe circumstances (60, 61). Therefore, we speculated that inflammation and visfatin have a close relationship and inhibiting the expression of endogenous visfatin may exacerbate the outbreak of inflammation. Through bidirectional control of pro-inflammatory and anti-inflammatory processes, our prior research demonstrated that visfatin might be essential for maintaining the body’s stability both in the physiological condition and in the acute inflammatory state generated by LPS (14). This supports the idea that endogenous visfatin exhibits a dual regulatory role on inflammation in the physiological condition and in the acute inflammatory state generated by LPS.

### The regulation effects of exogenous visfatin on apoptosis in RAW264.7 cells

Apoptosis controls inflammatory cytokines in inflammatory reaction to speed up the inflammatory process (62). Numerous studies have confirmed a connection between apoptosis and visfatin in a variety of cell types, including B or T cells, vascular smooth muscle cells and macrophages (19–22). Visfatin’s ability to prevent apoptosis in a sepsis model is extraordinary (63). According to a study, visfatin may prevent apoptosis in the juvenile ovary by increasing Bcl-2 expression (64). Furthermore, the effects of visfatin seems also to rely on dosage and the state of cellular activation, another study suggested that visfatin promotes the apoptosis of endothelial progenitor cells *via* the induction of pro-inflammatory cytokines through the NF-κB pathway (10). Hence, our findings first supported exogenous visfatin’s ability to prevent LPS-induced apoptosis in RAW264.7 cells.

Currently, it is known that stimuli cause Caspase-9 and Caspase-3 to be activated by the aberrant expression of Bcl-2 and Bax, which leads to caspase-3 eventually inducing apoptosis (37, 65). Visfatin inhibits apoptosis by reducing the rate of apoptosis in alveolar epithelial cells and by maintaining the production of autophagic factors through activation of the PI3K-AKT signaling pathway, whereas Bcl-2 gene family members primarily regulate apoptosis (66). Additionally, findings from our earlier work exhibited that visfatin increases apoptosis in rat spleen as evidenced by the lowered level of Bcl-2 and the elevated amounts of Bax, Caspase-3 and a decreased Bcl-2/Bax ratio as well as the reduced levels of Bcl-2 and Bcl-2. Exogenous visfatin, on the other hand, might prevent lymphocytes exposed to LPS from going into apoptosis, as evidenced by the increased levels of Bcl-2 and Bcl-2/Bax ratio and decreased levels of Bax and Caspase-3 mRNA (14).

### The regulation effects of endogenous visfatin on apoptosis in mice immune organs

In this work, it was shown that visfatin expression was blocked, and that this resulted in a large rise in the expression of the anti-apoptotic genes Bcl2L1 and Bcl2a1a and a significant decrease in the expression of the pro-apoptotic genes Fas, Bax, and Caspase3. According to this finding, when cells were exposed to an acute inflammatory response caused by LPS, apoptosis rose dramatically and the response to apoptosis was inhibited by decrease of endogenous visfatin expression in the spleen and thymus of mice. However, in the spleen and thymus of mice exposed to LPS, visfatin expression was considerably reduced. In line with this, several studies have confirmed that visfatin has dual functions both intracellular and extracellular (13, 21, 67). And endogenous visfatin plays diverse roles in pathophysiology, potentially mediating both adaptive and maladaptive responses (15). Additionally, abnormal upregulation of endogenous visfatin may aggravate cells which were severely damaged by oxidative stress, ultimately leading to cell dysfunction and apoptosis (68).

As a result, we hypothesized that endogenous visfatin might have a variety of functions, largely based on the body’s physiological condition. Acute inflammation brought on by LPS can cause endogenous visfatin to express itself to an abnormally high degree, which could result in apoptosis. Additionally, we looked into the apoptotic pathway that visfatin modifies and found both intrinsic and extrinsic routes. According to the findings, under LPS-induced inflammatory conditions, visfatin may boost Bcl2l1 and Bcl2a1a expressions while preventing the production of Fas. Consequently, in line with the finding of our earlier research (66), hence, it is proposed that, exogenous visfatin may conduct a dual control mechanism on cell apoptosis by stimulating it in a non-inflammatory state and suppressing it in an inflammatory one *via* the death-receptor and mitochondrial apoptotic pathways.

## Conclusion

Exogenous visfatin exhibits dual effects on inflammation throughout the inflammatory response by modulating IL-1α, TNFRSF1B, and LIF, expression levels as well as taking part in a number of signaling networks, including the MAPK and Rap1 signaling pathways. Additionally, in mice treated with LPS, inhibiting endogenous visfatin might worsen the immunological organs’ inflammatory reaction and even result in rapid mortality.

In terms of apoptosis, exogenous visfatin can prevent apoptosis in RAW264.7 cells by modulating the expression of Bcl2l1, Bcl2a1a, and Fas as well as taking part in multiple signaling networks, including the PI3K/AKT and Hippo signaling pathways. However, endogenous visfatin encourages apoptosis in mice immune organs *via* controlling the amounts of Bcl2l1, Fas, Caspase3, Bcl2a1a, and Bax expression.

## Data availability statement

The datasets presented in this study can be found in online repositories. The names of the repository/repositories and accession number(s) can be found below: https://www.ncbi.nlm.nih.gov/, PRJNA293603.

## Ethics statement

The animal study was reviewed and approved by The Scientific Ethic Committee of Huazhong Agricultural University approved animal experiments (HZAUMO-2019-025).

## Author contributions

HS conceived the study. Z-WZ and KX performed all the experiments with the help of SW, X-YN, W-JY, M-QL, ZY, and W-HZ. Z-WZ and KX analyzed all the data with the help of SW. Z-WZ drafted the manuscript with help of KX and ARA. HS, W-CB, and ZR helped in writing the manuscript and proofing reading. All authors contributed to the article and approved the submitted version.

## Funding

National Natural Science Fund Project of China (No.31772687), Fundamental Research Funds for the Central Universities (No.2662020DKPY001) and the Opening Subject of Key Laboratory of Prevention and Control Agents for Animal Bacteriosis, Ministry of Agriculture and Rural Affairs and Hubei Provincial Key Laboratory of Animal Pathogenic Microbiology (KLPCAAB-2021-02) supported the current study.

## Conflict of interest 

Author W-HZ was employed by Wuhan Keqian Biology Company Limited.

The remaining authors declare that the research was conducted in the absence of any commercial or financial relationships that could be construed as a potential conflict of interest.

## Publisher’s note

All claims expressed in this article are solely those of the authors and do not necessarily represent those of their affiliated organizations, or those of the publisher, the editors and the reviewers. Any product that may be evaluated in this article, or claim that may be made by its manufacturer, is not guaranteed or endorsed by the publisher.
