# Supplementary material for: Visfatin is a multifaceted molecule that exerts regulation effects on inflammation and apoptosis in RAW264.7 cells and mice immune organs
Source: Front Immunol. 2022 Dec 1;13:1018973. doi: 10.3389/fimmu.2022.1018973 (PMC9753570; doi:10.3389/fimmu.2022.1018973)
Supplement: Supplementary file 1 [file Table_1.docx]

| States  Groups | Living | | | | Early apoptotic | | | | Late apoptotic | | | | Necrotic | | | |
| --- | --- | --- | --- | --- | --- | --- | --- | --- | --- | --- | --- | --- | --- | --- | --- | --- |
|  | 6 h | 12 h | 18 h | 24 h | 6 h | 12 h | 18 h | 24 h | 6 h | 12 h | 18 h | 24 h | 6 h | 12 h | 18 h | 24 h |
| Control | 89  ±2 | 85  ±1.7 | 82  ±0.1 | 86  ±3.1 | 2.7  ±0.7 | 2.6  ±1.4 | 0.9  ±0.1 | 2.7  ±0.9 | 5  ±1.3 | 10.6  ±0.9 | 10.4  ±0.2 | 6.7  ±1.4 | 3.1  ±0.2 | 2  ±0.7 | 6.7  ±0.1 | 4.9  ±1 |
| Visfatin | 91  ±0.6 | 86  ±2.3 | 69  ±8.4 | 61  ±6.6  ** | 3.0  ±0.5 | 3.0  ±1.5 | 1.6  ±0.9 | 8.1  ±1 | 3.9  ±0.4 | 10  ±1.2 | 15.4  ±2.4 | 4.5  ±1.5  ** | 2.4  ±0.4 | 0.8  ±0.2 | 8.7  ±0.6 | 26  ±5.8  ** |
| LPS | 69  ±12 | 57  ±3.8 | 50  ±3.9 | 31  ±5.8 | 14.2±5 | 7.1  ±1.6 | 6.6  ±1.8 | 6.3  ±1 | 10.1±2.7 | 23.6±3.4 | 14.8  ±0.5 | 16  ±1.8 | 4.7  ±2.4 | 12  ±1.2 | 28.5  ±5 | 47  ±4.9 |
| LPS+  visfatin | 76  ±8 | 64±2.3  * | 45  ±3.1 | 30  ±3.4 | 7.2  ±1  * | 6.8  ±0.5 | 4.8  ±0.7 | 5.9  ±1  * | 6  ±1.6  * | 18.5  ±3.5* | 20.6  ±0.5** | 12.9  ±0.5 | 11  ±6.7 | 10.4±2 | 29  ±3 | 50  ±5 |

**Supplementary Table 1: The result of flow cytometry assay**

**P*<0.05, ***P*<0.01
